# Gender Differences in Job Burnout, Career Choice Regret, and Depressive Symptoms Among Chinese Dental Postgraduates: A Cross-Sectional Study

**DOI:** 10.3389/fpubh.2022.832359

**Published:** 2022-04-27

**Authors:** Li Yan, Xiaogang Zhong, Lu Yang, Huiqing Long, Ping Ji, Xin Jin, Li Liu

**Affiliations:** ^1^School of Public Health and Management, Chongqing Medical University, Chongqing, China; ^2^NHC Key Laboratory of Diagnosis and Treatment on Brain Functional Diseases, The First Affiliated Hospital of Chongqing Medical University, Chongqing, China; ^3^Key Laboratory of Psychoseomadsy, Stomatological Hospital of Chongqing Medical University, Chongqing, China; ^4^Chongqing Stomatological Association, Chongqing, China

**Keywords:** job burnout, career choice regret, depressive symptoms, dental postgraduates, gender differences

## Abstract

**Background:**

Job burnout, career choice regret, and depressive symptoms among medical students have received widespread attention. However, little is known about the role of gender in these areas for dental postgraduates. This study aimed to explore gender differences in job burnout, career choice regret, and depressive symptoms among Chinese dental postgraduates.

**Methods:**

The data were collected from an epidemiological survey conducted by our group from February 2021 to March 2021. We used a self-administered questionnaire covering demographic characteristics, the Maslach Burnout Inventory, the Primary Care Evaluation of Mental Disorders scale, and the Career Choice Regret scale. Univariate and multivariable analyses were performed to explore influencing factors.

**Results:**

A total of 558 valid dental postgraduate questionnaires were included in this study. The prevalence of job burnout, career choice regret, and depressive symptoms exceeded 30% in males and females. The prevalence of job burnout was 4.7% higher in females than in males; career choice regret was 12.2% higher in females than in males (*P* < 0.05), and depressive symptoms were 4.9% higher in females than in males. The multivariable analysis showed that factors associated with job burnout for males were sleep time and career choice regret for females. The influencing factors on career choice regret for females were postgraduate entrance examination score, sleep time, and job burnout. Depressive symptoms were a common influencing factor for job burnout and career choice regret in male and female dental postgraduates. Also, job burnout and depressive symptoms had the highest odds ratio for influencing factors on each other.

**Conclusion:**

Over 30% of dental postgraduates suffered from job burnout, career choice regret, and depressive symptoms, and incidences were higher in females. A discrepancy of influencing factors existed between male and female dental postgraduates. Targeted measures should be taken to change this situation.

## Introduction

Job burnout originates from the individual's long-term response to chronic emotional and interpersonal stress at work or their psychological response to work-related stress (1). There are three major features of job burnout: physical and emotional fatigue caused by work-related stressors, negative attitude toward career, and feeling disconnected from others (2). Studies have shown that job burnout is widespread in medical students (3). For example, the prevalence of job burnout among medical students in the United States was reported to be 52.8% (4), and it ranged from 38.1% to 53.8% for Chinese medical students (5).

Regret is a typical emotion related to decision-making, and career choice regret is experienced when the obtained career is not what the student expected or hoped for. Numerous studies have shown that career choice regret is strongly associated with job burnout in medical students (6–8). A survey from China reported that career choice regret was the strongest risk factor for job burnout in neurology postgraduates. A possible explanation is that regret induced a more intense dislike of the outcome of the career choice, which could increase the risk of burnout (9). Furthermore, career choice regret has been associated with depressive symptoms (10). Research shows that experiencing regret is related to an increase in depressive tendencies, the explanation being that regret plays a partial mediation role between other variables and depression (11). Evidence from neuroimaging also suggests that when regret occurs, brain regions associated with depression are activated, suggesting that regret may trigger depressive emotions (9).

In China, a qualified dental postgraduate needs at least 8 years of training. During this period, they face a huge academic burden, strict clinical requirements, and serious stress. Previous studies have demonstrated that medical students have more stress, more burnout, and a greater prevalence of mental health disorders than dental students (12–14). Therefore, research on dental postgraduates also applies to the whole medical student population. Dental postgraduates are engaged in clinical work, serve as part of the country's medical workforce, and participate in a wide range of complex tasks, such as treating patients and performing examinations (15). The use of sharp instruments may cause adverse complications or accidental injury to patients (7). In addition, dental postgraduates have to deal with anxious patients who are afraid of pain related to dental treatment (16). Their academic work involves an extensive educational curriculum and examinations. They are also under severe pressure to publish articles to meet the requirements for graduation. They have many sources of stress in their daily lives, including lack of sleep and leisure time, economic dependence, and the challenge of balancing family and work (2, 17–19). Chronic exposure to these stress factors may lead to job burnout, career choice regret, and depressive symptoms (15). Furthermore, job burnout and depression often intensify each other (20). Therefore, it is necessary to explore the relationship between these stress factors and job burnout, career choice regret, and depressive symptoms.

Among the numerous demographic characteristics, gender plays a key role in job burnout, career choice, and depressive symptoms (21–23). For example, a survey from Pakistan found that female medical undergraduates were more likely to experience depression than males (24). Another study from a large sample size in China also found that career choice regret was more prevalent among female medical students (6). A survey from Saudi Arabia found that gender was a significant predictor of stress and anxiety among undergraduate dental students, with females having a significantly higher prevalence of stress and anxiety than males (25). These differences can be explained, to some extent, by the gender role theory. Traditionally, women tend to express their fatigue (higher overload) at work, while men usually do not express their feelings (26). Although previous studies have found that gender has an important effect on job burnout, career choice regret, and depressive symptoms, most of the studies included gender as an independent variable (6, 24, 25). Furthermore, some of these results were controversial (23, 27, 28). Finally, there is a lack of gender-specific comparison of Chinese dental postgraduates.

The current evidence prompts the following questions: Do Chinese dental postgraduates, in general, have job burnout, career choice regret, and depressive symptoms? If yes, how serious is the situation? Is there a gender difference in job burnout, career choice regret, and depressive symptoms among Chinese dental postgraduates? What are the associated factors of job burnout, career choice regret, and depressive symptoms between Chinese male and female dental postgraduates, respectively? To address the above issues, our group conducted a cross-sectional survey. We hope that this study can provide a reference for related research and lead to the implementation of follow-up intervention measures.

## Materials and Methods

### Data Collection

It is an accepted rule of thumb that when the events per variable(EPV) exceeded 10, the regression model is considered to be stable (2, 29), and the minimum sample size in this study was calculated to be 130 dental postgraduates. A convenience sampling method was used to recruit the participants. The research group conducted a survey from February 2021 to March 2021 to explore the status of job burnout, career choice regret, and depressive symptoms among Chinese dental postgraduates. First, the members of the research group contacted the directors of the College of Stomatology and dental postgraduates in dental departments in local hospitals *via* WeChat, telephone, or email, and invited them to participate in this survey. Second, when directors agreed to participate, postgraduates were invited by their directors to participate in this study. The inclusion criteria are as follows: Chinese dental students; from master's degree to doctorate level. To ensure the accuracy of the data, we evaluated the quality of all questionnaires, according to the following exclusion criteria: the same answer was given for all the questionnaire items; If two or more consecutive questionnaires have identical answers for the same hospital or college, only one questionnaire is included.

### Survey Questionnaire

The questionnaire was constructed on the questionnaire star platform (https://www.wjx.cn/), a free platform widely used for surveys in China, which creates an internet link to the questionnaire. The members of the research group sent the questionnaire to the directors who agreed to participate in this survey. The directors then invited the postgraduates in their study groups to participate in this survey. We adjusted the questionnaire settings as follows: To reduce the extent of missing data, only fully completed questionnaires could be submitted. Participants were prompted to complete unanswered questions; To ensure that each questionnaire was unique, only one questionnaire from an internet protocol address was accepted.

The questionnaire consisted of five parts. The first part was the front page of the questionnaire. It introduced the background and purpose of the survey, informed participants of their rights and potential risks, and informed them that the survey was voluntary and anonymous.

The second part was the demographic characteristics, including gender, age, academic year, type of degree, family income per month, postgraduate entrance examination score, work or study time per week, daily sleep time, marital status, whether have children, and who had ever undertaken part-time job during studies.

The third part included a self-administrated Chinese version of the Maslach Burnout Inventory, a self-reported 22-item questionnaire that measures the frequency and intensity of job burnout in the allied health professions (30). This instrument measured three domains, emotional exhaustion (9 items, score 0–54), depersonalization (5 items, score 0–30), and personal accomplishment (8 items, score 0–48). Each item was rated on a 7-point Likert-like scale (range 0–6). Scores between 19 and 26 for emotional exhaustion, six and nine for depersonalization, and 34 and 39 for personal accomplishment were considered to reflect an average level of job burnout (31). Emotional exhaustion (with a score of ≥27) or depersonalization (with a score of ≥10) indicate a high degree of job burnout (4, 31, 32). This scale has been successfully used with Chinese neurologists and postgraduates (6, 31).

The fourth part was the Primary Care Evaluation of Mental Disorders (PRIME-MD), consisting of two questions. If the responder answered “Yes” to at least one of the following two questions, they were considered to have depressive symptoms: “Have you often been bothered by feeling down, depressed, or hopeless during the past month?” and “Have you often been bothered by having little interest or pleasure in doing things during the past month?” PRIME-MD has been widely used to screen for depressive symptoms (4, 33), and the performance of this scale is similar to that of longer instruments (34), with a sensitivity of 86 to 96% and a specificity of 57 to 75% for depressive symptoms (34, 35).

The last part included a question about career choice regret: “If you could go back, would you choose to be a doctor again?” “Yes,” “Not sure,” and “No” were the provided options, and “No” indicated career choice regret (36). This form has been used in many previous studies to assess the degree of career choice regret (6, 36, 37).

### Statistical Analysis

We performed all analyses with SPSS version 25.0 (IBM Corp., Armonk, NY). Categorical variables were summarized as frequencies and percentages, while continuous variables were summarized as means with SD or medians with interquartile ranges depending on the data distribution. First, a chi-square test was adopted to explore whether the demographic information of male and female dental postgraduates is comparable. Second, a chi-square test was adopted to evaluate the overall job burnout, depressive symptoms, and career choice regret between the male and female dental postgraduates. Third, a chi-square test or Fisher exact test was adopted to explore the influencing factors of job burnout, depressive symptoms, and career choice regret. Finally, to further screen the influencing factors, binary logistic regression (forward elimination, backward elimination, and enter model) was adopted for the statistically significant influencing factors from the univariate analysis. The variables showed multicollinearity if the variance inflation factor (VIF) was larger than 10. Statistically, significance was defined as *P* < 0.05.

## Results

### Participant Characteristics

A total of 580 questionnaires were collected, and 22 were excluded because the same answer was given for all the questionnaire items. This left 558 valid questionnaires (effective response rate: 96.2%) for subsequent analysis. The Cronbach's α coefficient of the Maslach Burnout Inventory-22 was 0.75, with emotional exhaustion being 0.90, personal accomplishment being 0.86, and depersonalization being 0.43. The Cronbach's α coefficient of the PRIME-MD was 0.867.

Of the respondents, 69.2% (386/558) were female, and 30.8% (172/558) were male. Most of the respondents were 22–25 years old, had clinical practice degrees, slept 6–8 h a day, had no children, and had not undertaken part-time work. The degree type was statistically different between males and females (*P* < 0.05). The ratio of clinical practice degrees among males is higher than among females. There was no statistical gender difference in other demographic factors, indicating that the demographic information between male and female dental postgraduates is comparable. Table 1 shows the demographic variables of gender comparisons.

**Table 1:** Demographic characteristics of participants.

**Variables**	**Male**	**Female**	***χ^2^***	***P***
	**N (%)**	**N (%)**		
Age (years)			0.544	0.762
22–25	100 (58.1)	230 (59.6)		
26–30	63 (36.6)	141 (36.5)		
>30	9 (5.3)	15 (3.9)		
Academic year			2.702	0.440
First-year, master's degree	74 (43.1)	141 (36.5)		
Second-year, master's degree	44 (25.5)	102 (26.4)		
Third-year, master's degree	46 (26.7)	117 (30.3)		
Doctor's degree	8 (4.7)	26 (6.7)		
Degree type			4.265	0.039
Academic practice	40 (23.3)	123 (31.8)		
Clinical practice	132 (76.7)	263 (68.2)		
Family income (RMB per month)			0.909	0.635
<5,000	67 (38.9)	135 (34.9)		
5,000–10,000	63 (36.6)	155 (40.3)		
>10,000	42 (24.5)	96 (24.8)		
Postgraduate entrance examination score			0.002	0.999
<330	52 (30.2)	116 (30.1)		
330–360	68 (39.5)	153 (39.6)		
>360	52 (30.3)	117 (30.3)		
Work or study time per week (h)			1.173	0.556
<45	60 (34.8)	128 (33.3)		
45–55	47 (27.3)	123 (31.8)		
>55	65 (37.9)	135 (34.9)		
Daily sleep time (h)			0.628	0.731
<6	30 (17.4)	61 (15.8)		
6–8	132 (76.7)	307 (79.6)		
>8	10 (5.9)	18 (4.6)		
Marital status			1.031	0.597
Single	83 (48.2)	204 (52.8)		
Partner	75 (43.6)	152 (39.5)		
Married	14 (8.2)	30 (7.7)		
Whether have children			0.388	0.534
No	164 (95.4)	363 (94.0)		
Yes	8 (4.6)	23 (6.0)		
Who had ever undertaken part-time job			0.256	0.613
No	147 (85.5)	336 (87.0)		
Yes	25 (14.5)	50 (13.0)		

### Prevalence Comparison of Job Burnout, Career Choice Regret, and Depressive Symptoms

The results show that 42.5% of female dental postgraduates reported job burnout, compared to 37.8% of males, but this difference was not significant (*P* = 0.298). Female dental postgraduates had a higher prevalence of emotional exhaustion (*P* = 0.017), a lower prevalence of depersonalization (*P* = 0.469), and a higher prevalence of low personal accomplishment than their male counterparts (*P* = 0.282). For career choice regret, the prevalence was 33.1% and 45.3% for male and female dental postgraduates, respectively, and the difference was statistically significant (*P* < 0.05). For depressive symptoms, 45.6% of female dental postgraduates reported depressive symptoms compared to 40.7% of males, but there was no statistical significance (*P* = 0.282), as Table 2 shows.

**Table 2:** Prevalence comparison of job burnout, career choice regret, and depressive symptoms.

**Items**	**Male**	**Female**	***χ^2^***	***P***
	**N (%)**	**N (%)**		
Job burnout	65 (37.8)	164 (42.5)	1.085	0.298
Emotional exhaustion	33 (19.2)	111 (28.8)	5.692	0.017
Depersonalization	51 (29.7)	103 (26.7)	0.524	0.469
Personal accomplishment	70 (40.7)	176 (45.6)	1.158	0.282
Career choice regret	57 (33.1)	175 (45.3)	7.287	0.007
Depressive symptoms	70 (40.7)	176 (45.6)	1.158	0.282

### Univariate Analysis of Job Burnout, Career Choice Regret, and Depressive Symptoms

The univariate analysis showed that factors associated with job burnout for males were daily sleep time, career choice regret, and depressive symptoms; for females, the factors were career choice regret and depressive symptoms. Career choice regret for males was associated with job burnout and depressive symptoms; for females, it was associated with postgraduate entrance examination scores, daily sleep time, job burnout, and depressive symptoms. Depressive symptoms in males and females were both associated with job burnout and career choice regret (all *P* < 0.05), as Table 3 shows.

**Table 3:** Univariate analysis of job burnout, career choice regret, and depressive symptoms.

	**Job burnout**				**Career choice regret**				**Depressive symptoms**			
	**Male (%)**	***χ^2^***	**Female (%)**	***χ^2^***	**Male (%)**	***χ^2^***	**Female (%)**	***χ^2^***	**Male (%)**	***χ^2^***	**Female (%)**	***χ^2^***
Age (years)	*P* = 0.776	0.546	*P* = 0.581	1.085	*P* = 0.427	1.924	*P* = 0.894	0.225	*P =* 0.513	1.394	*P* = 0.903	0.204
22–25	39 (39)		102 (44.3)		35 (35)		102 (44.3)		42 (42.4)		105 (45.7)	
26–30	22 (34.9)		57 (40.4)		21 (33.3)		66 (46.8)		25 (39.7)		65 (46.1)	
>30	4 (44.4)		5 (33.3)		1 (1.8)		7 (46.7)		2 (22.2)		6 (40)	
Academic year	*P* = 0.757	1.204	*P* = 0.626	1.748	*P* = 0.367	3.174	*P* = 0.106	6.115	*P* = 0.669	1.581	*P* = 0.058	7.493
First-year, master's degree	28 (37.8)		57 (40.4)		29 (39.1)		53 (37.5)		31 (41.9)		52 (36.9)	
Second-year, master's degree	19 (43.2)		42 (41.2)		14 (31.8)		50 (49)		16 (36.4)		54 (52.9)	
Third-year, master's degree	16 (34.8)		51 (43.6)		11 (23.9)		57 (48.7)		21 (45.7)		56 (47.9)	
Doctor's degree	2 (25)		14 (53.8)		3 (37.5)		15 (57.6)		2 (25.0)		14 (53.8)	
Degree type	*P* = 0.057	3.627	*P* = 0.700	0.148	*P* = 0.103	2.663	*P* = 0.171	1.872	*P* = 0.402	0.701	*P* = 0.281	1.163
Academic	10 (25)		110 (41.8)		9 (22.5)		62 (50.4)		14 (35.0)		61 (49.5)	
Clinical	55 (41.7)		54 (43.9)		48 (36.3)		113 (42.9)		56 (42.4)		115 (43.7)	
Family income (RMB per month)	*P* = 0.098	4.636	*P* = 0.370	1.987	*P* = 0.486	1.443	*P* = 0.299	2.416	*P* = 0.923	0.160	*P* = 0.112	4.383
<5,000	28 (41.8)		51 (37.8)		25 (37.3)		68 (50.3)		28 (41.8)		59 (43.7)	
5,000–10,000	27 (42.9)		71 (45.8)		21 (33.3)		64 (41.2)		26 (41.3)		80 (51.6)	
>10,000	10 (23.8)		42 (43.8)		11 (26.1)		43 (44.7)		16 (38.1)		37 (38.5)	
Postgraduate entrance examination score	*P* = 0.091	4.804	*P* = 0.485	1.446	*P* = 0.870	0.279	*P* = 0.007	9.994	*P* = 0.583	1.079	*P* = 0.299	2.414
<330	22 (42.3)		49 (42.2)		16 (30.7)		51 (43.9)		24 (46.2)		53 (45.6)	
330–360	19 (27.9)		70 (45.8)		24 (35.2)		83 (54.2)		25 (36.8)		76 (49.6)	
>360	24 (46.2)		45 (38.5)		17 (32.6)		41 (35)		21 (40.4)		47 (40.1)	
Work or study time per week (h)	*P* = 0.125	4.160	*P* = 0.064	5.491	*P* = 0.236	2.889	*P* = 0.646	0.875	*P* = 0.243	2.832	*P* = 0.945	0.113
<45	25 (41.7)		57 (44.5)		23 (38.3)		54 (42.1)		24 (40.0)		59 (46.0)	
45–55	12 (25.5)		42 (34.1)		11 (23.4)		59 (47.9)		15 (31.9)		57 (46.3)	
>55	28 (43.1)		65 (48.1)		23 (35.3)		62 (45.9)		31 (47.7)		60 (44.4)	
Daily sleep time (h)	*P* <0.001	15.409	*P* = 0.069	5.360	*P* = 0.168	3.546	*P* = 0.001	13.483	*P* = 0.101	4.569	*P* = 0.050	5.990
<6	21 (70)		34 (55.7)		14 (46.6)		40 (65.5)		17 (56.7)		36 (59.0)	
6–8	41 (31.1)		122 (39.7)		39 (29.5)		125 (40.7)		48 (36.4)		134 (43.6)	
>8	3 (30)		8 (44.4)		4 (40.0)		10 (55.5)		5 (50)		6 (33.3)	
Marital status	*P* = 0.974	0.053	*P* = 0.788	0.477	*P* = 0.258	2.741	*P* = 0.408	1.795	*P* = 0.248	2.803	*P* = 0.959	0.084
Single	32 (38.6)		90 (44.1)		27 (32.5)		89 (43.6)		39 (47.0)		94 (46.0)	
Partner	28 (37.3)		62 (40.8)		28 (37.3)		69 (45.3)		27 (36.0)		69 (45.3)	
Married	5 (35.7)		12 (40)		2 (14.2)		17 (56.6)		4 (28.6)		13 (43.3)	
Whether have children	*P* = 0.131	2.283	*P* = 0.228	1.454	*P* = 0.041	4.159	*P* = 0.497	0.461	*P* = 0.355	0.857	*P* = 0.521	0.412
No	64 (39)		157 (43.3)		57 (34.7)		163 (44.9)		68 (41.5)		167 (46.0)	
Yes	1 (12.5)		7 (30.4)		0		12 (52.1)		2 (25.0)		9 (39.1)	
Who had ever undertaken part-time job	*P* = 0.255	1.297	*P* = 0.590	0.290	*P* = 0.294	1.103	*P* = 0.187	1.740	*P* = 0.605	0.267	*P* = 0.503	0.449
No	53 (36.1)		141 (42)		51 (34.6)		148 (44.0)		61 (41.5)		151 (44.9)	
Yes	12 (48)		23 (46)		6 (24)		27 (54.0)		9 (36.0)		25 (50.0)	
Job burnout	–		–		*P* = 0.013	6.210	*P* <0.001	18.238	*P* <0.001	18.804	*P* <0.001	76.195
No	–		–		28 (26.1)		80 (36.0)		30 (28.0)		59 (26.5)	
Yes	–		–		29 (44.6)		95 (57.9)		40 (61.5)		117 (73.1)	
Career choice regret	*P* = 0.013	6.210	*P* <0.001	18.238	–		–		*P* = 0.001	10.447	*P* <0.001	20.783
No	79 (68.7)		69 (32.7)		–		–		37 (32.2)		74 (35.0)	
Yes	29 (50.8)		95 (54.2)		–		–		33 (57.9)		102 (58.2)	
Depressive symptoms	*P* <0.001	18.804	*P* <0.001	76.195	*P* = 0.001	10.447	*P* <0.001	20.783	–		–	
No	26 (25.2)		47 (22.3)		25 (24.2)		73 (34.7)		–		–	
Yes	39 (56.5)		117 (66.4)		32 (46.3)		102 (57.9)		–		–	

*P (probability, value according to Chi-square test)*.

### Multivariable Analysis of Job Burnout, Career Choice Regret, and Depressive Symptoms

Career choice regret and daily sleep time were the job burnout risk factors for female and male dental postgraduates, respectively. A gradual increase in sleep time decreased the risk of job burnout for males. The risk factors for career choice regret in females were the postgraduate entrance examination scores, job burnout, and daily sleep time. Depressive symptoms were the common risk factors for males and females in job burnout and career choice regret. Table 4 shows that job burnout and career choice regret were the common risk factors for depressive symptoms in males and females (*P* < 0.05). Multicollinearity analysis results indicated no severe collinearity between variables in job burnout, career choice regret, and depressive symptoms. The detailed results of logistic regression and VIF are shown in the Supplementary Table.

**Table 4:** Logistic regression of job burnout, career choice regret and depressive symptoms.

**Variables**	**Job burnout**				**Career choice regret**				**Depressive symptoms**			
	**Male**		**Female**		**Male**		**Female**		**Male**		**Female**	
	***OR (95% CI)***	***P***	***OR (95% CI)***	***P***	***OR (95% CI)***	***P***	***OR (95% CI)***	***P***	***OR (95% CI)***	***P***	***OR (95% CI)***	***P***
Postgraduate entrance examination score	–	–	–	–	–	–	–	0.008	–	–	–	–
<330	–	–	–	–	–	–	1 (Reference)		–	–	–	–
330–360	–	–	–	–	–	–	1.58 (0.94–2.65)	0.082	–	–	–	–
>360	–	–	–	–	–	–	0.70 (0.4–1.22)	0.210	–	–	–	–
Daily sleep time (h)	–	0.003	–	–	–	–	–	0.003	–	–	–	–
<6	1 (Reference)		–	–	–	–	1 (Reference)		–	–	–	–
6–8	0.22 (0.09–0.54)	0.001	–	–	–	–	0.37 (0.20–0.68)	0.001	–	–	–	–
>8	0.17 (0.03–0.87)	0.033	–	–	–	–	0.74 (0.24–2.31)	0.610	–	–	–	–
Job Burnout	NA	–	NA	–	–	–	1.71 (1.07–2.74)	0.025	3.68 (1.89–7.19)	<0.001	6.21 (3.93–9.81)	<0.001
Career choice regret	–	–	1.82 (1.15–2.87)	0.011	NA	–	NA	–	2.46 (1.23–4.89)	0.011	2.02 (1.28–3.18)	0.003
Depressive symptoms	3.87 (1.95–7.65)	<0.001	6.21 (3.93–9.81)	<0.001	2.90 (1.51–5.58)	0.001	1.93 (1.21–3.08)	0.006	NA	–	NA	–

‘*_', indicates these variables were excluded in regression models; OR, odds ratio; P (probability, value according to logistic regression analysis); NA, variable was not included in the logistic model*.

## Discussion

In this study, a self-administered questionnaire was adopted to investigate the gender differences in job burnout, career choice regret, and depressive symptoms among Chinese dental postgraduates. We explored the prevalence and influencing factors of job burnout, career choice regret, and depressive symptoms between male and female dental postgraduates. To our knowledge, this is the first study to examine this issue in this population.

The incidence of job burnout in our sample exceeded 40%, higher than the rate reported for dental students in Saudi Arabia (30.14%) and in Spain (26%) (38, 39), suggesting that it is more prevalent in this population. A contributing factor may be that the dental students in our survey are receiving postgraduate education. Under the Chinese medical education system, a qualified dental postgraduate needs at least 8 years of training. In addition, the emotional exhaustion of females was significantly higher than that of their male counterparts. This is consistent with previous studies (40, 41). A possible explanation is that females are more likely to express their emotional distress, are more sensitive to stress, and are more vulnerable to negative emotions than males (42–44). Multivariable analysis showed that daily sleep time was an influencing factor in job burnout among male dental postgraduates; the longer the daily sleep time, the lower the risk of job burnout. A possible explanation is that lack of sleep will lead to physical fatigue and the inability to cope with work effectively (45). Career choice regret was the influencing factor for female dental postgraduates. This factor was consistent with the results of previous surveys. A ranking of the things Americans regret most identified education and career as the most likely areas for regret (46). Research on regret behavior also shows that regret can lead to the desire to correct mistakes, undo events, and get a second chance. Regret can also lead to a greater aversion to the outcome of choices made (9). Career choice regret could induce an aversion to the chosen career, thereby increasing the risk of job burnout.

The incidence of career choice regret is significantly higher in females, consistent with another survey of Chinese healthcare students (47). A possible explanation is that females are expected to be more likely to prioritize their family and children. At the same time, the enormous stress of graduate education can easily lead to conflicts between family and study, which may increase their career choice regret (48). Multivariable analysis showed that postgraduate entrance examination score, daily h of sleep, and job burnout are associated with career choice regret for female dental postgraduates. Graduate entrance exam scores have a strong effect on being allocated a supervisor, especially for those with scores of 330–360 (not good and not bad). One possible explanation is that expectations are high for this group. However, their results tend to be worse than expected, potentially triggering a strong emotional response and increasing their career choice regret. Too much or too little sleep may increase the risk of career choice regret. Long-term chronic sleep deprivation is an adverse circumstance that can severely reduce medical students' career identity, significantly impacting career choice (49). However, excessive sleep duration can also cause psychological problems (50–52), affecting the efficiency of postgraduates' work and study, thereby reducing their career identity. Our findings also indicate that consistent with a previous study (53), dental postgraduates with job burnout are more likely to experience career choice regret.

We found depressive symptoms to be a common risk factor for job burnout and career choice regret among male and female dental postgraduates. This finding was consistent with previous studies. For example, a study from China found that medical students who experienced depressive symptoms were more likely to drop out of medical school (54). Another survey found that the presence of depression was the variable predicting high emotional exhaustion, one of the main components of job burnout (55). A possible explanation is that the symptoms of job burnout overlapped with some psychiatric symptoms, particularly mood exhaustion and depressive symptoms (56). Depressive symptoms were also a risk factor for low medical career interest and high intention to leave (8, 57).

Our results show that the prevalence of depressive symptoms is higher in females, and it is not statistically significant. However, several studies have shown that gender is an influential factor in depressive symptoms (58–60). For example, a survey of Chinese high school students found that girls are more likely to suffer from depressive symptoms due to academic stress. The possible explanation is that girls generally respond to stressors with higher levels of depressive symptoms than boys (61). Our research found that the factors associated with depressive symptoms, namely job burnout and career choice regret, were similar between males and females. Previous studies found that job burnout was a significant risk factor for depression in medical students (62, 63). Research has also found that work-related risk factors for job burnout were predictors of depression (64). Furthermore, a study of Chinese neurology postgraduates demonstrated an overlap between job burnout and depressive symptoms (10). Job burnout, career choice regret, and depressive symptoms may overlap because depressive symptoms are the risk factor with the highest odds ratio for job burnout and career choice regret, and job burnout and career choice regret are also the risk factor with the highest odds ratio for depressive symptoms.

As females are becoming increasingly active in the healthcare industry, this disparity may have a significant impact. Appropriate measures are urgently needed to reduce the incidence of job burnout, career choice regret, and depressive symptoms in dental postgraduates. Education departments should pay more attention to adjustments in dental postgraduates' curriculum and clinical work so that students can combine work with rest. University administrators should reform the way students choose tutors and should conduct comprehensive assessments, not just postgraduate entrance examination scores. There is also a need to strengthen the training of stress management for graduate students to improve the prevention of students' negative psychological issues. Finally, early screening for psychological problems of dental students should be strengthened, paying more attention to students who may have problems and helping them solve problems quickly.

## Conclusions

Dental postgraduates have a relatively high prevalence of job burnout, career choice regret, and depressive symptoms. Female dental postgraduates showed a higher prevalence and more risk factors than their male peers. The daily h of sleep and depressive symptoms were associated with increased job burnout levels for male dental postgraduates, and career choice regret and depressive symptoms were associated with increased job burnout levels for female dental postgraduates. Depressive symptoms were associated with increased career choice regret levels for male dental postgraduates, and postgraduate entrance examination scores, daily h of sleep, and depressive symptoms were associated with increased career choice regret levels for female dental postgraduates. Job burnout and career choice regret were associated with increased depressive symptom levels in male and female dental postgraduates. Appropriate measures are urgently needed to address the current situation.

## Limitations

This study has some limitations. First, this study used convenience sampling. Therefore, our data may be subject to selection bias. Second, some scales, such as PRIME-MD, involve recollections of events from a month ago, so there may be a recall bias. Third, this was a cross-sectional survey, so we could not determine whether the associations we found were causal. Fourth, due to space limitations, this study on the impact of gender differences is not sufficiently deep. Therefore, further studies using larger and more diversified sample sizes are needed to validate our results.

## Data Availability Statement

The original contributions presented in the study are included in the article/Supplementary Material, further inquiries can be directed to the corresponding author/s.

## Ethics Statement

Written informed consent was obtained from the individual(s) for the publication of any potentially identifiable images or data included in this article.

## Author Contributions

LYan and XZ proposed the concept and design. LYang, HL, and PJ collected the data. LYan analyzed and interpreted the data, and wrote the manuscript. XZ, HL, and LYang edited the manuscript. XJ and LL gave guidance on the content of the article and supervised the study. PJ and XJ provided the funding. All authors contributed to the article and approved the submitted version.

## Funding

This work was supported by the Natural Science Foundation Project of China (Grant Nos. 81870775 and 81500855).

## Conflict of Interest

The authors declare that the research was conducted in the absence of any commercial or financial relationships that could be construed as a potential conflict of interest.

## Publisher's Note

All claims expressed in this article are solely those of the authors and do not necessarily represent those of their affiliated organizations, or those of the publisher, the editors and the reviewers. Any product that may be evaluated in this article, or claim that may be made by its manufacturer, is not guaranteed or endorsed by the publisher.
